# Risk factors for multidrug resistance in pulmonary tuberculosis patients with diabetes mellitus

**DOI:** 10.3389/fmed.2025.1516207

**Published:** 2025-01-31

**Authors:** Lianpeng Wu, Na Chen, Dandan Xia, Xiangao Jiang

**Affiliations:** ^1^Department of Clinical Laboratory Medicine, The Ding Li Clinical College of Wenzhou Medical University, Wenzhou Central Hospital, Wenzhou, Zhejiang, China; ^2^Key Laboratory of Diagnosis and Treatment of New and Recurrent Infectious Diseases of Wenzhou, Wenzhou Sixth People’s Hospital, Wenzhou, Zhejiang, China; ^3^Department of Infectious Diseases, The Ding Li Clinical College of Wenzhou Medical University, Wenzhou Central Hospital, Wenzhou, Zhejiang, China

**Keywords:** tuberculosis, diabetes mellitus, multidrug-resistant tuberculosis, risk factors, prediction model

## Abstract

**Objective:**

This study aimed to investigate the risk factors for multidrug resistance (MDR) in patients with pulmonary tuberculosis (PTB) and diabetes mellitus (DM), including those with and without prior TB treatment.

**Methods:**

A retrospective study was conducted from 1 January 2021, to 31 December 2023, at Wenzhou Central Hospital. Patients diagnosed with PTB and DM were included, with multidrug-resistant tuberculosis (MDR-TB) defined as resistance to at least rifampicin and isoniazid. Data on demographics, clinical symptoms, laboratory tests, and treatment history were collected. Multivariate logistic regression analysis was used to identify independent risk factors for MDR, and receiver operating characteristic (ROC) curves were constructed to evaluate the predictive value of these factors.

**Results:**

A total of 318 patients were analyzed, with 253 in the non-MDR group and 65 in the MDR group. Significant independent predictors of MDR included a history of TB treatment, smoking, and elevated hemoglobin A1c (HbA1c) levels. ROC curve analysis showed that the combination of TB treatment history, smoking history, and HbA1c levels had an area under the curve (AUC) of 0.809, with 64.62% sensitivity and 82.61% specificity. In patients without prior TB treatment, smoking history and HbA1c were identified as independent risk factors, with an AUC of 0.771 for their combination. For patients with prior TB treatment, place of residence and pulmonary cavity were independent predictors, with an AUC of 0.802 for their combination.

**Conclusion:**

This study highlights the importance of smoking history, HbA1c levels, place of residence, and pulmonary cavity as risk factors for MDR in PTB and DM patients. Early identification of these risk factors can aid in the timely diagnosis and treatment of MDR-TB, potentially reducing its burden. Further research is needed to develop targeted interventions based on these findings.

## 1 Introduction

Tuberculosis (TB), the second-largest cause of death from a single source of infection globally after COVID-19, poses a significant burden on public health worldwide (1). The 2023 global tuberculosis report estimates 10.6 million TB cases and 1.3 million deaths in 2022, with an incidence rate of 133/100,000 (2). Global efforts are underway to accelerate the decline in TB incidence, aiming to achieve the strategic goal set by the World Health Organization to eliminate TB epidemic by 2035 (3). However, the high incidence of drug-resistant tuberculosis (DR-TB), especially multidrug-resistant tuberculosis (MDR-TB), poses a significant obstacle to achieving this goal, which is a worrying issue. Current data indicates that approximately 410,000 individuals were affected by DR-TB globally in 2022, with a treatment success rate of only 63% (2).

The increasing prevalence of diabetes mellitus (DM) presents a significant global health concern (4). According to the latest report from the International Diabetes Federation, it is estimated that 425 million individuals worldwide will have DM by 2023, with this number projected to rise to 783 million by 2045 (5). DM exacerbates the burden of TB, with previous research indicating that patients with DM are three times more likely to develop active TB (6). Global studies have attributed approximately 15% of TB cases to DM (7), while a study in China found that around 17% of TB cases were linked to DM (8). A study by Kong et al. (9) in Southwest China found that compared with pulmonary tuberculosis (PTB) patients, PTB patients with DM had no difference in delayed treatment, but the treatment success rate was significantly lower than that of PTB patients. Furthermore, DM has a certain impact on the emergence of MDR-TB. Various studies have demonstrated a positive correlation between DM and the incidence of MDR-TB (10, 11). MDR-TB is a form of TB that is resistant to at least rifampicin and isoniazid, making its treatment more challenging, prolonged, costly, and associated with higher rates of adverse reactions and lower cure rates compared to regular TB (12). As a nation grappling with both DM and MDR-TB burdens, China faces significant challenges in TB control (13). Early identification the risk factors for MDR-TB can help to curb the progression of MDR-TB, enable prompt diagnosis and treatment, and alleviate the burden of MDR-TB. The objective of this research is to investigate the risk factors for MDR in individuals suffering from PTB and DM, encompassing both patients who have never undergone TB treatment and those who have previously received such treatment, to provide a fundamental basis for the clinical diagnosis, therapeutic management, and preventive strategies against MDR-TB.

## 2 Materials and methods

### 2.1 Study design and subject selection

**FIGURE 1:**
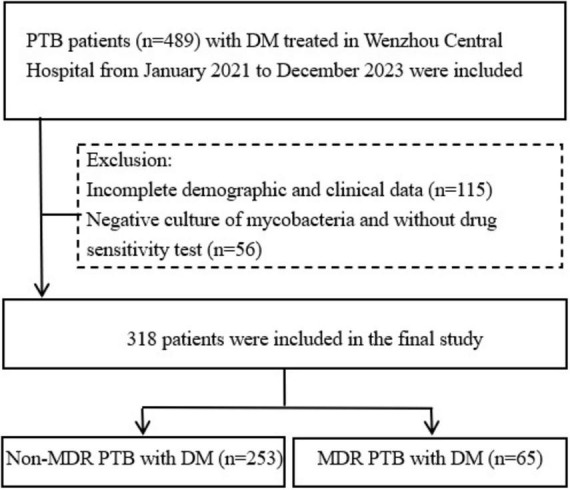
Flow chart of the patients included in the study. PTB, pulmonary tuberculosis; DM, diabetes mellitus; MDR, multidrug resistance.

This study follows the Strengthening the Reporting of Observational Studies in Epidemiology guidelines. The study was conducted from 1 January 2021, to 31 December 2023, at Wenzhou Central Hospital, and involved a retrospective analysis of all patients diagnosed with PTB and DM. The inclusion criteria of PTB were based on the Chinese diagnostic criteria of pulmonary tuberculosis (2017 Edition): (1) positive acid-fast smear in sputum; (2) positive culture of *Mycobacterium tuberculosis* (MTB) in sputum or bronchoalveolar lavage fluid (BALF); (3) positive nucleic acid test in sputum or BALF; (4) positive pathology in lung tissue. PTB can be diagnosed if one of the above four items is met. Patients with positive culture of MTB in sputum or BALF and drug sensitivity test were diagnosed as MDR-TB at least to rifampicin and isoniazid. The diagnostic criteria of DM were as follows: (1) fasting plasma glucose ≥7.0 mmol/L; (2) oral glucose tolerance test (75 g glucose) 2-h blood glucose ≥11.1 mmol/L; (3) HbA1c ≥ 6.5%. Exclusion criteria: (1) patients with incomplete demographic and clinical data; (2) patients with negative culture of mycobacteria and without drug sensitivity test. This study initially enrolled 489 patients with PTB and DM. However, 115 patients were excluded due to incomplete demographic and clinical data, and an additional 56 patients were excluded because they had negative mycobacterial cultures and no drug sensitivity testing. Consequently, the final analysis included 318 patients, who were categorized into two groups based on drug sensitivity test results: the non-MDR group, comprising 253 patients, and the MDR group, consisting of 65 patients (Figure 1).

### 2.2 Data collection

The case records of 489 patients were examined via the hospital’s case management system, and relevant data were retrospectively gathered. This included sociodemographic details such as age, gender, body mass index, marriage, place of residence, occupation, migration status, smoking and drinking history; baseline clinical symptoms like fever, cough, expectoration, hemoptysis, thoracodynia, chest tightness, fatigue, weight loss, and night sweats; information on combined with other diseases; results of laboratory tests; CT imaging examination; and clinical diagnosis.

### 2.3 Statistical analysis

Statistical analysis was performed using SPSS version 26.0. Continuous variables were first tested for normality using the Kolmogorov–Smirnov test. If the data did not conform to a normal distribution, they were expressed as medians and interquartile ranges, and comparisons between groups were made using the Mann–Whitney *U* test. Categorical variables were expressed as frequencies and percentages, and comparisons between groups were conducted using Pearson’s Chi-squared test, continuity-corrected Chi-squared test, or Fisher’s exact test. Variance inflation factor (VIF) was used for multiple collinear diagnosis of risk factors. Multidrug resistance risk factors were analyzed using multivariate logistic regression analysis. The predictive value of identified risk factors, both individually and in combination, for MDR-TB was evaluated using receiver operating characteristic (ROC) curves and area under the curve (AUC). A *P*-value < 0.05 was considered statistically significant.

## 3 Results

### 3.1 Characteristics of PTB and DM patients with MDR-TB and non-MDR-TB

The study included 318 patients with PTB and DM. Of these, 253 patients (79.56%) had non-MDR PTB with DM, while 65 patients (20.44%) had MDR PTB with DM. The two groups did not significantly differ in terms of gender, marriage situation, occupation, migrant status, or place of residence. In the non-MDR group, the median age was 62 years, compared to 53 years in the MDR group, showing a significant age difference (*P* < 0.05). Additionally, the median course of DM was significantly longer in the MDR group at 60 months, compared to 36 months in the non-MDR group (*P* < 0.05). There was no significant difference in the treatment of DM between the two groups. However, a higher percentage of patients in the MDR group had a history of TB treatment and smoking than those in the non-MDR group (*P* < 0.05). Additionally, there were no significant differences in the incidence of other diseases between the two groups. In terms of clinical manifestations, the MDR group exhibited a higher prevalence of pulmonary cavities compared to the non-MDR group, with no significant differences observed in other clinical symptoms. Levels of hemoglobin A1c (HbA1c) and fasting plasma glucose (FPG) were significantly elevated in the MDR group relative to the non-MDR group, whereas other laboratory test results did not exhibit significant differences. Specific parameters are detailed in Table 1.

**TABLE 1:** The demographic and clinical parameters of patients with PTB and DM.

Variables	Total *n* = 318 (%)	Non-MDR PTB with DM *n* = 253 (%)	MDR PTB with DM *n* = 65 (%)	*P*-value
Gender				0.264
Male	273 (85.85)	220 (86.96)	53 (81.54)	
Female	45 (14.15)	33 (13.04)	12 (18.46)	
Age (years)	60 (49–70)	62 (52–70)	53 (45–53)	0.001
Body mass index (BMI)	21.48 (19.38–23.66)	21.30 (19.37–23.67)	21.74 (19.47–23.55)	0.810
Marriage				0.376*
Unmarried	14 (4.40)	9 (3.56)	5 (7.69)	
Married	281 (88.37)	224 (88.54)	57 (87.69)	
Divorced	11 (3.46)	9 (3.56)	2 (3.08)	
Widowed	12 (3.77)	11 (4.34)	1 (1.54)	
Occupation				0.827
Unemployed	267 (83.96)	213 (84.19)	54 (83.08)	
Employed	51 (16.04)	40 (15.81)	11 (16.92)	
Migrant				
Yes	47 (14.78)	35 (13.83)	12 (18.46)	0.348
No	271 (85.22)	218 (86.17)	53 (81.54)	
Residence				0.070
Urban	164 (51.57)	137 (54.15)	27 (41.54)	
Rural	154 (48.43)	116 (45.85)	38 (58.46)	
Course of DM (month)	36 (1–120)	36 (0.2–120)	60 (12–120)	0.034
DM treatment modality				
Irregular treatment	101 (31.76)	82 (32.41)	19 (29.23)	0.098
Oral hypoglycemic agent	147 (46.23)	115 (45.45)	32 (49.23)	
Insulin	52 (16.35)	38 (15.02)	14 (21.54)	
Oral antidiabetic drugs + insulin	18 (5.66)	18 (7.12)	0 (0)	
History of TB treatment	51 (16.04)	25 (9.88)	26 (40.00)	<0.001
Smoking history	149 (46.86)	109 (43.08)	40 (61.54)	0.008
History of drinking	92 (28.93)	78 (30.83)	14 (21.54)	0.141
Coronary heart disease	19 (5.97)	16 (6.32)	3 (4.62)	0.822*
Chronic obstructive pulmonary disease	9 (2.83)	8 (3.16)	1 (1.54)	0.776*
Hypertension	125 (39.31)	104 (41.11)	21 (32.31)	0.195
Hyperuricemia	140 (44.03)	115 (45.45)	25 (38.46)	0.311
Cerebral infarction	22 (6.92)	19 (7.51)	3 (4.62)	0.585*
Respiratory failure	22 (6.92)	19 (7.51)	3 (4.62)	0.585*
Chronic kidney disease	21 (6.60)	14 (5.53)	7 (10.77)	0.216*
Solid tumor	12 (3.77)	10 (3.95)	2 (3.08)	1.000*
Cirrhosis	6 (1.89)	5 (1.98)	1 (1.54)	1.000*
Syphilis infection	6 (1.89)	5 (1.98)	1 (1.54)	1.000*
Hepatitis B infection	32 (10.06)	26 (10.28)	6 (9.23)	0.803
Hepatitis C infection	7 (2.20)	4 (1.58)	3 (4.62)	0.311*
HIV infection	2 (0.63)	1 (0.40)	1 (1.54)	0.368**
Fever	79 (24.84)	63 (24.90)	16 (24.62)	0.962
Cough	273 (85.85)	216 (85.38)	57 (87.69)	0.633
Expectoration	233 (73.27)	180 (71.15)	53 (81.54)	0.091
Hemoptysis	48 (15.09)	36 (14.23)	12 (18.46)	0.395
Thoracodynia	24 (7.55)	18 (7.11)	6 (9.23)	0.754*
Chest tightness	42 (13.21)	33 (13.04)	9 (13.85)	0,865
Fatigue	53 (16.67)	44 (17.39)	9 (13.85)	0.494
Emaciation	85 (26.73)	73 (28.85)	12 (18.46)	0.091
Night sweats	20 (6.29)	15 (5.93)	5 (7.69)	0.813*
Pulmonary cavity	206 (64.78)	154 (60.87)	52 (80.00)	0.004
Pleural effusion	79 (74.68)	64 (72.85)	15 (78.97)	0.712
HbA1c, %	8.3 (7.1–10.0)	8.0 (6.9–9.5)	10.0 (8.2–11.6)	<0.001
ESR, mm/h	43 (25–65)	43 (26–64)	43 (24–66)	0.906
WBC count, 10^9^/L	7.5 (5.8–9.5)	7.6 (5.9–9.5)	7.5 (5.6–9.7)	0.696
NEUT count, 10^9^/L	5.4 (3.9–7.0)	5.5 (3.9–7.0)	5.3 (4.0–6.9)	0.647
LYM count, 10^9^/L	1.3 (0.9–1.7)	1.2 (0.8–1.7)	1.5 (1.0–1.8)	0.111
HGB, g/L	123 (108–138)	123 (107–138)	124 (111–139)	0.633
PLT, 10^9^/L	267 (207–345)	267 (211–345)	267 (202–349)	0.725
ALT, U/L	17 (11–27)	16 (11–27)	19 (14–27)	0.199
ALB, g/L	33.4 (29.2–36.2)	33.1 (28.7–36.1)	34.0 (31.3–37.3)	0.062
FPG, mmol/L	7.9 (6.2–11.6)	7.3 (6.0–10.6)	10.5 (7.1–14.2)	<0.001
Cr, μmol/L	61 (52–76)	60 (52–76)	63 (51–76)	0.781
UA, μmol/L	308 (227–409)	306 (219–397)	338 (251–438)	0.090
CRP, mg/L	35.3 (16.2–77.6)	36.1 (12.5–78.6)	33.8 (20.5–69.8)	0.448
AFS				
Negative	174 (54.72)	141 (55.73)	33 (50.77)	0.473
Positive	144 (45.28)	112 (44.27)	32 (49.23)	

MDR, multidrug resistance; PTB, pulmonary tuberculosis; TB, tuberculosis; DM, diabetes mellitus; HbA1c, hemoglobin A1c; ESR, erythrocyte sedimentation rate; WBC, white blood cell; NEUT, neutrophil; LYM, lymphocyte; HGB, hemoglobin; PLT, platelet; ALT, alanine aminotransferase; ALB, albumin; FPG, fasting plasma glucose; Cr, creatinine; UA, uric acid; CRP, c-reactive protein; AFS, acid fast smear.

*By continuity correction Chi-square test.

**By Fisher’s exact test.

### 3.2 Univariate and multivariate analysis of risk factors for MDR in PTB and DM patients

The variables with statistical differences in univariate analysis (age, course of DM, history of TB treatment, smoking history, pulmonary cavity, HbA1c, and FPG) were diagnosed by multiple collinear diagnosis. The results showed that the tolerance was between 0.620 and 0.982, and the VIFs were less than 10, and there was no collinearity problem. The multivariate logistic regression analysis indicated that having a history of TB treatment, being a smoker, and having elevated HbA1c levels are significant independent predictors of MDR in individuals with PTB and DM, with specifics outlined in Table 2.

**TABLE 2:** Multivariate logistic regression analysis of risk factors of MDR in patients with PTB and DM.

Variable	β	SE	Wald	*P*-value	Odds ratio (95% CI)	VIF
Age	−0.022	0.014	2.270	0.132	0.979 (0.951–1.007)	1.368
Course of DM	0.003	0.002	1.726	0.189	1.003 (0.999–1.007)	1.118
History of TB treatment	2.040	0.390	27.371	0.000	7.688 (3.581–16.507)	1.019
Smoking history	0.719	0.336	4.591	0.032	2.053 (1.063–3.963)	1.154
Pulmonary cavity	0.341	0.396	0.743	0.389	1.406 (0.648–3.053)	1.059
HbA1c	0.331	0.091	13.138	0.000	1.393 (1.164–1.666)	1.612
FPG	0.020	0.047	0.177	0.674	1.020 (0.931–1.118)	1.435

TB, tuberculosis; DM, diabetes mellitus; HbA1c, hemoglobin A1c; FPG, fasting plasma glucose.

### 3.3 ROC curve analysis of predictive factors for MDR in PTB and DM patients

**FIGURE 2:**
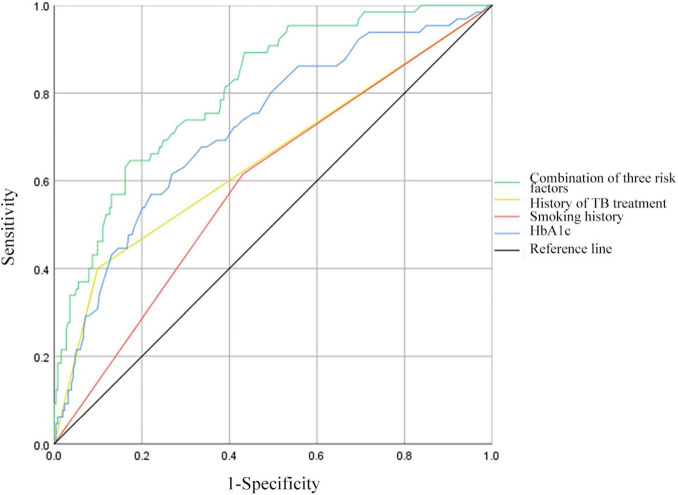
Receiver operating characteristic curves for history of TB treatment, smoking history, HbA1c, and all three risk factors combined for MDR in patients with PTB and DM. HbA1c, hemoglobin A1c.

Receiver operating characteristic curves were constructed to evaluate the predictive ability of TB treatment history, smoking history, HbA1c levels, and the combination of the above risk factors for MDR in patients with PTB and DM (Figure 2). For the history of TB treatment, the AUC was 0.651 (95% CI: 0.568–0.733), featuring a sensitivity of 40.00% and a specificity of 90.12%. Regarding smoking history, the AUC stood at 0.592 (95% CI: 0.515–0.669), with a sensitivity of 61.54% and a specificity of 56.92%. HbA1c demonstrated an AUC of 0.722 (95% CI: 0.652–0.793), with a sensitivity of 56.92% and a specificity of 76.28% at the optimal cut-off of 9.7%. Combining the history of TB treatment, smoking history, and HbA1c resulted in an AUC of 0.809 (95% CI: 0.753–0.865), with a sensitivity of 64.62% and a specificity of 82.61%.

### 3.4 Comparison of demographic and clinical characteristics in PTB and DM patients without previous TB treatment

Table 3 reveals that the median age for the non-MDR cohort was 62 years, whereas for the MDR cohort it was 50 years, with this age gap being statistically significant (*P* < 0.05). The MDR group also had a notably higher rate of smoking when compared to the non-MDR group (*P* < 0.05). In addition, the MDR group exhibited significantly elevated levels of HbA1c and FPG compared to the non-MDR group (*P* < 0.05). Other demographic and clinical characteristics did not show significant variations (*P* > 0.05, not listed).

**TABLE 3:** Comparison of demographic and clinical parameters in PTB patients with DM without a history of TB treatment.

Variables	Total *n* = 267 (%)	Non-MDR PTB and DM without a history of TB treatment *n* = 228 (%)	MDR PTB and DM without a history of TB treatment *n* = 39 (%)	*P*-value
Age (years)	60 (49–75)	62 (51–70)	50 (43–64)	<0.001
Smoking history	123 (46.07)	99 (43.42)	24 (61.54)	0.036
HbA1c, %	8.3 (7.2–10.0)	8.1 (7.0–9.6)	10.1 (9.1–11.6)	<0.001
FPG, mmol/L	7.8 (6.2–11.1)	7.4 (6.1–10.5)	10.4 (7.1–14.2)	0.001

MDR, multidrug resistance; PTB, pulmonary tuberculosis; DM, diabetes mellitus; HbA1c, hemoglobin A1c; FPG, fasting plasma glucose.

### 3.5 Univariate and multivariate analysis of risk factors for MDR in PTB and DM patients without previous TB treatment

The variables with statistical differences in univariate analysis (age, smoking history, HbA1c, and FPG) were diagnosed by multiple collinear diagnosis. The results showed that the tolerance was between 0.655 and 0.989, and the VIFs were less than 10, and there was no collinearity problem. Multivariate logistic regression analysis pinpointed smoking history and HbA1c as independent predictors of MDR in PTB and DM patients without previous TB treatment, with specifics in Table 4.

**TABLE 4:** Multivariate logistic regression analysis of risk factors of MDR in patients with PTB and DM without a history of TB treatment.

Variable	β	SE	Wald	*P*-value	Odds ratio (95% CI)	VIF
Age	−0.030	0.016	3.572	0.059	0.971 (0.941–1.001)	1.208
Smoking history	0.845	0.394	4.604	0.032	2.328 (1.076–5.037)	1.011
HbA1c	0.379	0.106	12.705	0.000	1.461 (1.186–1.801)	1.527
FPG	0.018	0.054	0.110	0.741	1.018 (0.916–1.132)	1.383

HbA1c, hemoglobin A1c; FPG, fasting plasma glucose.

### 3.6 ROC curve analysis of predictive factors for MDR in PTB and DM patients without previous TB treatment

**FIGURE 3:**
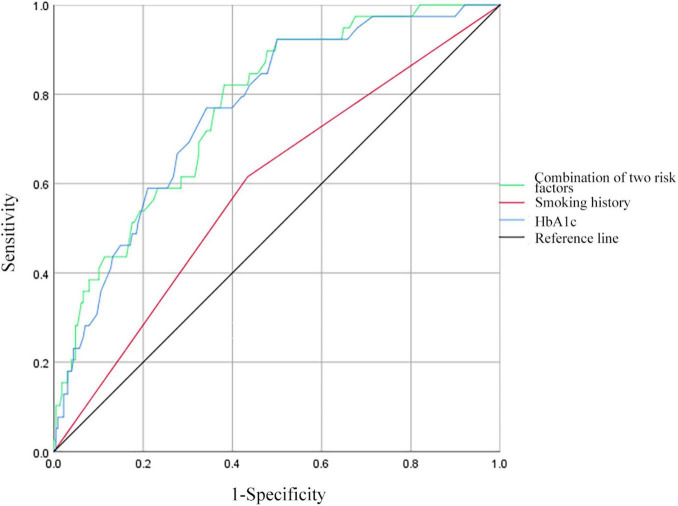
Receiver operating characteristic curves for smoking history, HbA1c, and all two risk factors combined for MDR in patients with PTB and DM without a history of TB treatment. HbA1c, hemoglobin A1c.

Receiver operating characteristic curves were constructed to evaluate the predictive ability of smoking history, HbA1c levels, and the combination of the above risk factors for MDR in patients with PTB and DM without previous TB treatment (Figure 3). For smoking history, the AUC was 0.591 (95% CI: 0.495–0.687), with 61.54% sensitivity and 56.58% specificity. HbA1c showed an AUC of 0.764 (95% CI: 0.689–0.839), with 76.92% sensitivity and 65.79% specificity at an optimal cut-off of 9.1%. Combining smoking history and HbA1c resulted in an AUC of 0.771 (95% CI: 0.699–0.844), with 82.05% sensitivity and 62.84% specificity.

### 3.7 Comparison of demographic and clinical parameters in PTB and DM patients with prior TB treatment

As per the data presented in Table 5, a notable distinction was observed in the prevalence of rural inhabitants, with the MDR cohort displaying a markedly elevated percentage in contrast to the non-MDR cohort, achieving statistical significance (*P* < 0.05). Furthermore, when it came to the incidence of expectoration and the presence of pulmonary cavities, the MDR group outpaced the non-MDR group, also with a significant statistical difference (*P* < 0.05). In addition, the levels of HbA1c and FPG were found to be considerably higher in the MDR group as opposed to the non-MDR group, once again reaching a significant level (*P* < 0.05). No significant differences were observed in other demographic and clinical parameters (*P* > 0.05, not listed).

**TABLE 5:** Comparison of demographic and clinical parameters in PTB patients with DM with a history of TB treatment.

Variables	Total *n* = 51 (%)	Non-MDR PTB and DM with a history of TB treatment *n* = 25 (%)	MDR PTB and DM with a history of TB treatment *n* = 26 (%)	*P*-value
Residence				0.003
Urban	26 (50.98)	18 (72.00)	8 (30.77)	
Rural	25 (49.02)	7 (28.00)	18 (69.23)	
Expectoration	38 (74.51)	15 (60.00)	23 (88.46)	0.020
Pulmonary cavity	37 (72.55)	13 (52.00)	24 (96.00)	0.001
HbA1c, %	8.1 (6.8–10.9)	7.6 (6.7–9.3)	9.8 (7.7–11.5)	0.017
FPG, mmol/L	8.6 (5.9–13.4)	6.9 (5.3–13.0)	11.8 (7.1–14.3)	0.033

MDR, multidrug resistance; PTB, pulmonary tuberculosis; DM, diabetes mellitus; HbA1c, hemoglobin A1c; FPG, fasting plasma glucose.

### 3.8 Univariate and multivariate analysis of risk factors for MDR in PTB and DM patients with prior TB treatment

The variables with statistical differences in univariate analysis (residence, expectoration, pulmonary cavity, HbA1c, and FPG) were diagnosed by multiple collinear diagnosis. The results showed that the tolerance was between 0.584 and 0.877, and the VIFs were less than 10, and there was no collinearity problem. Multivariate logistic regression analysis revealed that place of residence and pulmonary cavity as independent predictors for MDR in PTB and DM patients with prior TB Treatment, with specifics in Table 6.

**TABLE 6:** Multivariate logistic regression analysis of risk factors of MDR in patients with PTB and DM with a history of TB treatment.

Variable	β	SE	Wald	*P*-value	Odds ratio (95% CI)	VIF
Residence						1.207
Urban	–	–	–	–	1.00	
Rural	1.465	0.738	3.941	0.047	4.328 (1.019–18.385)	
Expectoration	1.227	0.937	1.715	0.190	3.412 (0.543–21.423)	1.248
Pulmonary cavity	2.182	0.943	5.359	0.021	8.868 (1.397–56.280)	1.141
HbA1c	0.153	0.199	0.588	0.443	1.165 (0.789–1.721)	1.650
FPG	0.073	0.111	0.434	0.510	1.076 (0.865–1.338)	1.713

HbA1c, hemoglobin A1c; FPG, fasting plasma glucose.

### 3.9 ROC curve analysis of predictive factors for MDR in PTB and DM patients with prior TB treatment

**FIGURE 4:**
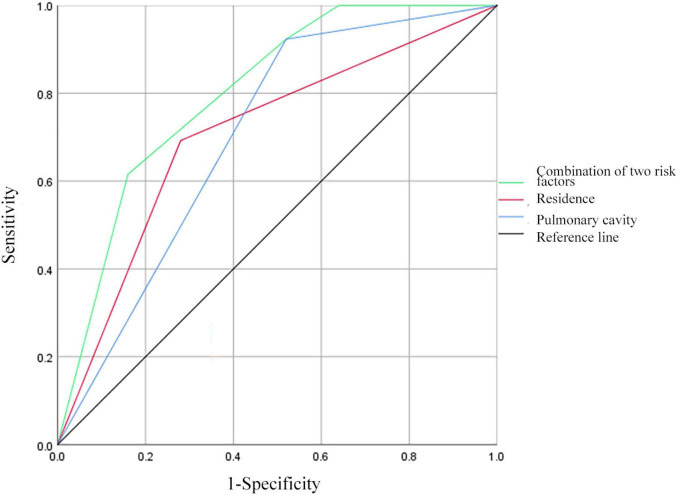
Receiver operating characteristic curves for residence, pulmonary cavity, and all two risk factors combined for MDR in patients with PTB and DM with a history of TB treatment.

Receiver operating characteristic curves were constructed to evaluate the predictive ability of residence, pulmonary cavity, and the combination of the above risk factors for MDR in patients with PTB and DM with prior TB treatment (Figure 4). The AUC for residence was 0.706 (95% CI: 0.560–0.852), demonstrating a sensitivity of 69.23% and a specificity of 72.00%. For pulmonary cavity, the AUC was 0.702 (95% CI: 0.554–0.849), with a sensitivity of 92.31% and a specificity of 48.00%. When combining residence and pulmonary cavity, the AUC increased to 0.802 (95% CI: 0.680–0.923), with a sensitivity of 61.54% and a specificity of 84.00%.

## 10 Discussion

The global burden of TB and DM is significant, with a prevalence rate of 16% worldwide (7). DM can lead to immune system dysfunction, alterations in cytokine levels, and activation of macrophages (14). Additionally, DM can result in higher mycobacterial loads, changes in the pharmacokinetics of anti-TB medications, and reduced treatment adherence, thereby increasing the risk of MDR-TB (11). Previous research indicates that the risk of MDR-TB is 2.1 to 8.8 times higher in TB patients with DM (15). The prevalence of MDR-TB among patients with DM ranges from 10% to 30% (16). Gaining insight into the clinical manifestations of TB and DM, along with the risk factors for MDR, plays a vital role in reducing the likelihood of encountering MDR-TB.

The research found that, when contrasted with patients who did not have MDR-TB, those with MDR-TB and DM had a younger median age, a greater frequency of prior TB treatment, a higher rate of smoking, a more common presence of pulmonary cavities, a longer duration of DM, as well as increased levels of HbA1c and FPG. Discrepancies exist in prior research regarding the correlation between age and MDR-TB, with some studies indicating older patients (17–19) and others suggesting younger ones (20, 21). Consistent with previous findings, TB treatment history, smoking history, and pulmonary cavity were identified as risk factors for multidrug resistance in this study (22, 23). The research by Fisher-Hoch et al. (24) also supports the notion that inadequate blood glucose control is closely linked to the development of MDR-TB, aligning with the outcomes of this study.

Prior research has predominantly concentrated on pinpointing the risk factors for MDR-TB in patients, yet there has been a scarcity of studies examining individuals who have both PTB and DM. Utilizing multivariate analysis, our investigation singled out HbA1c levels as an independent risk factor for MDR in patients suffering from PTB and DM. Specifically, we observed a higher predictive value for MDR when HbA1c level reached 9.7%. Different researchers have suggested varying HbA1c thresholds for predicting MDR, with some proposing 7% (25) and others 9.3% (26). HbA1c contributes to drug resistance through mechanisms such as inducing tissue hypoxia and oxidative stress, leading to isoniazid resistance (27, 28). Elevated HbA1c levels can prolong sputum culture conversion time and heighten the risk of MDR (29). Moreover, hyperglycemia can impact the absorption and metabolism of anti-TB drugs, potentially resulting in MDR-TB (30).

The research identified that having a smoking history acts as an independent risk factor for MDR-TB in individuals with PTB and DM. Other research studies have also reached similar conclusions (31, 32). The underlying mechanism is attributed to smoking-induced ciliary dysfunction, which compromises the mucous membrane and immune defenses of the respiratory tract, consequently elevating the susceptibility to primary MDR-TB infection (33). DR-TB arises through two pathways: transmitted (or primary) resistance, where individuals contract strains already resistant to drugs, and acquired (or secondary) resistance, which emerges when MTB strains mutate randomly in their genome and face selective pressure from anti-TB medications during treatment (34). It is commonly acknowledged that a past TB treatment experience is the primary driver of acquired drug resistance in TB patients and stands as a significant independent risk factor for MDR-TB (35). Our study’s results highlight that a history of TB treatment serves as an independent risk factor for MDR in patients with PTB and DM.

Prior studies have not thoroughly explored the risk factors for MDR in PTB patients with DM who either have not previously received TB treatment or have a history of such treatment. We carried out an in-depth analysis of the MDR risk factors in both groups. Our findings indicated that among patients with PTB and DM who had not been treated for TB before, smoking history and HbA1c levels remained independent risk factors for MDR. In contrast, for those with a history of TB treatment, factors like place of residence and the presence of pulmonary cavities were identified as independent risk factors for MDR. This analysis implies that medical supervision in rural regions might be inadequate, causing patients to either not complete their prescribed treatment or use non-standard anti-TB medications, which in turn raises the risk of developing MDR-TB (36). Pulmonary cavities provide a sealed and protective niche for MTB, enabling the bacteria to survive and proliferate within the host. Such an environment may aid MTB in adapting to and resisting anti-TB drugs, thus increasing the probability of MDR-TB emergence (37).

In research conducted by Li et al. (26), variables such as age under 65 years, HbA1c levels, and TB treatment history were utilized to predict the occurrence of MDR-TB in patients with TB and DM. The results indicated that the model achieved an AUC value of 0.878. Furthermore, when age under 65 years and HbA1c were combined to predict MDR-TB in patients with TB and DM who had previously been treated for TB, the model’s AUC reached 0.920. Another study by Lyu et al. (25) utilized HbA1c, age, erythrocyte sedimentation rate, hemoglobin, and C-reactive protein to predict MDR-TB occurrence in patients with TB and DM, resulting in a model AUC of 0.754. In the present study, the factors predictive of MDR-TB incidence in patients with PTB and DM were a history of TB treatment, smoking, and HbA1c, with the model’s AUC value being 0.809. Additionally, smoking and HbA1c were utilized to predict MDR-TB in patients with PTB and DM who had no prior TB treatment history, resulting in a model AUC of 0.771. Lastly, place of residence and the presence of a pulmonary cavity were the factors used to predict MDR-TB in patients with PTB and DM who had a history of TB treatment, with the model’s AUC value being 0.802. Collectively, these results indicate that the aforementioned models possess predictive utility.

This study has several limitations: (1) being retrospective, there may be residual confounding factors; (2) data were obtained from a single medical institution, which limits generalizability; (3) although cases of PTB with DM were collected from 2021 to 2023, the sample size is limited, particularly in patients with MDR-TB. Subsequent investigations ought to encompass a forward-looking study that engages various healthcare facilities. By doing so, the scope of research variables can be expanded, thereby facilitating a deeper probe into the risk factors associated with MDR in individuals afflicted with PTB and DM. Such an approach would pave the way for a more accurate and dependable basis upon which clinical decisions can be made.

## 5 Conclusion

To sum up, in patients suffering from PTB and DM, the history of TB treatment, smoking history, and HbA1c levels have been pinpointed as independent risk factors for MDR. When these three factors are combined, they exhibit predictive significance for MDR within this specific patient group. For those PTB and DM patients without a prior TB treatment history, smoking history and HbA1c stand out as independent risk factors for MDR. Conversely, in PTB and DM patients who have undergone TB treatment, place of residence and the presence of a pulmonary cavity emerge as independent risk factors for MDR. These distinctions in risk factors underscore the necessity of acknowledging the diversity in clinical diagnoses and merit careful consideration in medical practice.

## Data Availability

The raw data supporting the conclusions of this article will be made available by the authors, without undue reservation.
